# Hyper-Ballistic Superdiffusion of Competing Microswimmers

**DOI:** 10.3390/e26030274

**Published:** 2024-03-21

**Authors:** Kristian Stølevik Olsen, Alex Hansen, Eirik Grude Flekkøy

**Affiliations:** 1 Institut für Theoretische Physik II—Weiche Materie, Heinrich-Heine-Universität Düsseldorf, D-40225 Düsseldorf, Germany; 2 Nordita, Royal Institute of Technology and Stockholm University, Hannes Alfvéns väg 12, 23, SE-106 91 Stockholm, Sweden; 3 PoreLab, Department of Physics, Norwegian University of Science and Technology, NO-7491 Trondheim, Norway; alex.hansen@ntnu.no; 4 PoreLab, The Njord Centre, Department of Physics, University of Oslo, NO-0316 Oslo, Norway; e.g.flekkoy@fys.uio.no; 5 PoreLab, Department of Chemistry, Norwegian University of Science and Technology, NO-7491 Trondheim, Norway

**Keywords:** anomalous diffusion, porous media, microswimmers

## Abstract

Hyper-ballistic diffusion is shown to arise from a simple model of microswimmers moving through a porous media while competing for resources. By using a mean-field model where swimmers interact through the local concentration, we show that a non-linear Fokker–Planck equation arises. The solution exhibits hyper-ballistic superdiffusive motion, with a diffusion exponent of four. A microscopic simulation strategy is proposed, which shows excellent agreement with theoretical analysis.

## 1. Introduction

The transport of self-propelled particles through an obstacle-laden or porous environments is of importance to a broad range of scientific disciplines [1]. With applications ranging from the dispersion of contaminants in soils to transport of cells inside the body, and even medical applications in the context of micro- and nano-robotics, such processes provide a fruitful and important avenue of research from theoretical, experimental and industrial perspectives.

Transport of passive particles, such as Brownian colloids or tracers, through disordered porous media have been studied intensively in recent decades, and is, to a large extent, understood through theories such as the framework presented by Brenner [2]. Biological swimmers, on the other hand, display a plethora of phenomena not seen in the passive counterparts, most of which arise from their *active* nature, i.e., their ability to absorb energy from the environment [3]. In the majority of cases, it is assumed that this energy is used to produce directed *persistent* motion, after which it is dissipated back into the environment. This break both the classical fluctuation-dissipation theorem and detailed balance, making such systems non-equilibrium and fundamentally different from passive systems [4]. Self-propelled motion in porous media is an active area of research, displaying many intriguing phenomena such as enhanced motion and optimal swimming strategies [5,6,7,8,9,10,11], directional locking [12,13], and hydrodynamic trapping at obstacles [14,15].

Persistent motion of self-propelled particles is known to be the origin of many interesting phenomena in active systems. One example is the motility-induced accumulation of particles near solid obstacles or surfaces. Such accumulation can be purely dynamical in origin, where the finite-time correlations in the particle direction of motion give rise to extended durations, whereby a particle collides with the solid [1,16,17,18]. In porous media, this may give rise to long durations of trapping in dead-ends of the porous matrix. Combined with other effects of geometric confinement present in media with a high filling fraction, this can give rise to anomalous diffusion of the sub-diffusive type, whereby the particles mean square displacement scales in time as $\langle r^{2}\left(t\right)\rangle ∼t^{2\tau }$, with 0≤τ≤1/2 [19]. This is typically attributed to power-law distributed trapping times, and is common for both passive and active systems in strongly heterogeneous environments [20,21,22,23,24,25,26].

Superdiffusion with 1/2≤τ≤1 can also be observed, with typical examples including Lévy flights, animal migration patterns and tracers in turbulent flows [27,28,29,30,31]. Hyper-ballistic diffusion, on the other hand, where τ≥1, is much more rare. Hyper-ballistic diffusion seems almost like a contradiction, since it suggests transport that is faster than motion without any change in the direction of motion. There are still a few systems that exhibit such behavior. The random acceleration process is a model where $\langle r^{2}\left(t\right)\rangle ∼t^{3}$, which has seen many applications [32]. For example, it can be mapped onto the motion of a semi-flexible polymer inside a tube [33]. Quantum interference effects give Gaussian distribution with $\langle r^{2}\left(t\right)\rangle ∼t^{3}$ [34,35]. Such effects have also been observed in optical experiments studying wave packets moving through random potentials [36], an effect which is related to Anderson localization [37]. Both in classical and quantum systems, hyper-ballistic transport may occur for particles that receive unbounded amounts of energy from some random potential. For example, Golubovic et al. finds superdiffusion with $\langle r^{2}\left(t\right)\rangle ∼t^{18/8}$ in a time-dependent random potential [38]. A simple random walk model with memory effects, called the “elephant random walk” because elephants have long memories, also gives rise to superdiffusion, but with sub-ballistic behavior [39].

One way in which hyper-ballistic diffusion can appear is when the particle velocity is allowed to grow without bounds [40]. For normal Langevin systems, such behavior is not possible, since the fluctuation-dissipation theorem ensures a balance between the fluctuations driving the system and the dissipation. For active systems, such as biological microswimmers, the fluctuation-dissipation theorem is famously broken, making these systems potential candidates for hyper-ballistic diffusion. In this paper, we propose a simple model where self-propelled swimmers move in a porous media while competing for resources. Hyper-ballistic motion is shown to arise as a direct consequence of a nutrient landscape that dynamically changes with the swimmer density.

## 2. Model of Competing Swimmers

**Figure 1:**
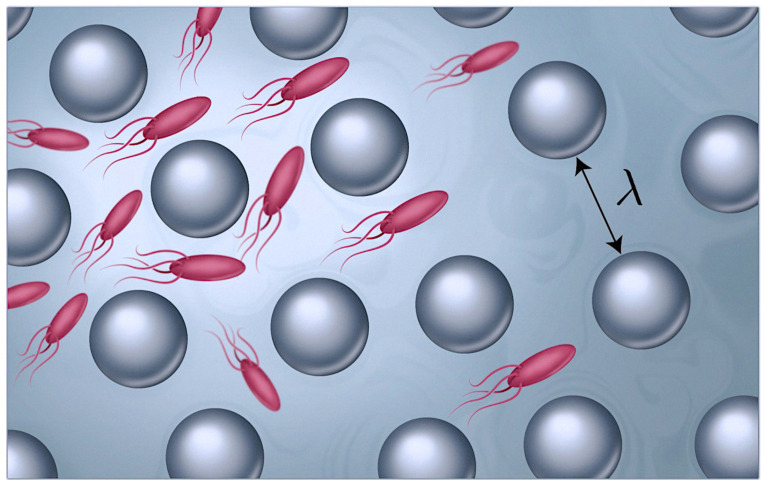
Swimmers in a porous medium with a characteristic pore size λ. Lighter colors indicate nutrition depletion.

The *N* swimmers move in an isotropic porous medium that defines a mean free path length λ (see Figure 1). The swimmers are assumed to move in straight lines at a speed v(x,t) until they hit the walls of the porous media. As mentioned above, some cells generate more persistent straight-lined motion when starved or in the absence of external signals [41,42]. When the swimmers collide with the walls of the medium, after a characteristic distance λ, they randomly change direction. The porous medium is only represented in this minimal way, so that the effect of potential trapping in dead ends or near obstacle surfaces is ignored [19].

The swimmers also move through a nutrient concentration $C_{N}\left(x,t\right)$ which can change dynamically in time due to the swimmers presence. The study of swimmers motion in various chemical gradients, *chemotaxis*, has been studied for decades in the biological and biophysical communities [43,44]. The motion of cells may also change drastically under starved conditions, when the nutrient concentration is low. While some cells respond to such conditions by deactivating flagellar motors [45], other observations include more persistent straight-lined motion [41,42]. More recent studies have modeled nutrient or activity landscapes implicitly as time- or space-dependent self-propulsion speeds in models of active matter [46,47,48,49]. Recently, a dynamical resource landscape was considered in a lattice model [50].

In the present context, we consider in the simplest case an inverse relationship $C_{N}\left(x,t\right)$∼1/C(x,t) between the nutrient concentration $C_{N}\left(x,t\right)$ and swimmer concentration C(x,t), implying that the presence of many swimmers deplete the nutrients in that region. For example, the nutrients can be supplied at a given rate from the walls in the medium, for example due to prior accumulation. We shall take the pore size to be much smaller than the nutrient diffusion length, so that the nutrient supply is limited by the release rate from the walls. Each swimmer eats at a rate $∼C_{N}\left(x,t\right)$ and we shall assume that the nutrient supply is almost depleted, so that $C_{N}\left(x,t\right)$ is much smaller than the saturation limit, hence the competition. In a given pore volume, the food consumption will thus be $∼C_{N}\left(x,t\right)C\left(x,t\right)$. This consumption is balanced by the constant supply from the walls so that the nutrient concentration scales as $C_{N}\left(x,t\right)∼1/C\left(x,t\right)$, as above.

Since the swimmers move against a Stokes drag force ∝v, the power they dissipate by swimming is proportional to $v^{2}$. Assuming that they convert a constant fraction of the energy they consume into this power, we arrive at a swimming velocity scaling as $v^{2}\left(x,t\right)∼C_{N}\left(x,t\right)$. A similar scaling is also found in microscopic models for active particles where energy consumption is explicitly modeled [51,52,53]. Combining the above, we arrive at

(1) $$\left|v\left(C\right)\right|\equiv v\left(C\right)=v_{0}{\left(\frac{C}{C_{0}}\right)}^{-1/2}$$

where $v_{0}$ is the velocity at some reference concentration $C_{0}$. In other words, the swimmers will accelerate once their concentration is down and the food competition is reduced. Their motion is diffusive in the sense that swimmers perform a random walk with a concentration dependent step length λ. This concentration dependence represents an interaction with the neighboring swimmers that define the local concentration.

## 3. Simulation Model

**Figure 2:**
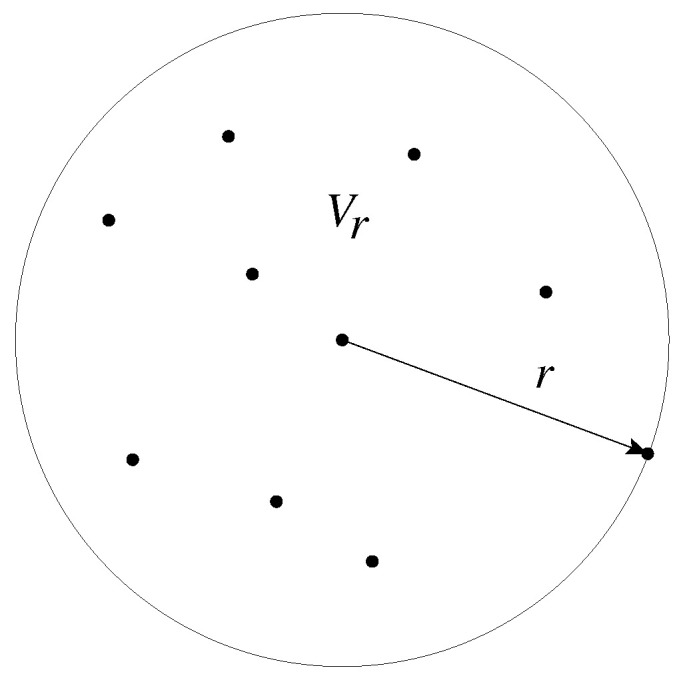
The volume $V_{r}$ from which the concentration is calculated. Here, $N_{r}=$ 10.

In order to simulate the collective motion of the swimmers, we represent them as point particles with positions $x_{i}$, i=1,…N that are updated according to the algorithm

(2) $$\begin{array}{cc} x_{i}\left(t+dt\right) & =x_{i}\left(t\right)+v\left(C\left(x_{i}\right)\right)dt \end{array}$$

(3) $$\begin{array}{cc} v_{i}\left(t+dt\right) & =\left\{\begin{array}{cc} Rv(C\left(x_{i}\right)) & if\Delta x_{i}>\lambda ,\\ v(C\left(x_{i}\right)) & otherwise, \end{array}\right. \end{array}$$

where dt is fixed small timestep, R is a rotation operator representing a random collision that uniformly re-orients the swimmers direction of motion, and $\Delta x_{i}$ is the displacement, since the last application of the random rotation operator R. We emphasize that, in the above algorithm, the velocity vector $v(C\left(x_{i}\right))$ only depends on the local concentration through its magnitude through Equation (1). The random re-orientations due to collisions only affect the swimmers direction of motion. To sample the local concentration around particle *i*, we use a scheme similar to that in Ref. [40]. Here, a number of interaction partners $N_{r}$ is introduced, along an associated volume $V_{r}\left(x\right)$ of a sphere that contains the $N_{r}$ nearest neighbors. This is illustrated in Figure 2. We emphasize that this type of interaction is non-local, in the sense that the range of interaction has no bounds. The local concentration used in Equations (2) and (3) is then sampled as

(4) $$C\left(x_{i}\left(t\right)\right)=\frac{N_{r}}{V_{r}\left(x_{i}\left(t\right)\right)}$$

The operator R in Equation (3) rotates $v_{i}$ into a new arbitrary direction without changing the speed, and the distance $\Delta x_{i}$ is calculated from this point, so that the next application of R may be determined. So, the swimmers are random walkers with a step length λ, and potentially, they spend several time steps to move λ along straight lines.

### 3.1. The Fokker–Planck Equation

Following the classical methods [54,55], we derive the Fokker–Planck equation that governs the time evolution of the particle concentration. The general form of the master equation takes the standard form

(5) $$\frac{\partial C(x,t)}{\partial t}=\int d^{3}r\left[C(x-r,t)W(x-r,r)-C(x,t)W(x,-r)\right]\;,$$

where W(x,r) is the jump rate associated with a step x→x+r. In the present case, the size of a step a swimmer takes depends on the nutrient concentration and, hence, also on the particle concentration. Assuming a slowly varying spatial dependence in the jump rates W(x−r,r), we may Taylor expand around x in its first argument. This results in the Fokker–Planck equation

(6) $$\frac{\partial C(x,t)}{\partial t}=\frac{1}{2}\int d^{3}rr_{i}r_{j}\frac{\partial^{2}}{\partial x_{i}\partial x_{j}}\left[C(x,t)W(x,r)\right]\;,$$

Rearranging the derivatives, we have

(7) $$\frac{\partial C(x,t)}{\partial t}=\frac{1}{2}\nabla^{2}\left[a_{2}\left(x\right)C\left(x,t\right)\right]\;,$$

where $a_{2}\left(x\right)$ is the second moment of the jump length per time,

(8) $$a_{2}\left(x\right)=\int d^{3}r\frac{r^{2}}{3}W\left(x,r\right)=\frac{1}{3}\frac{\lambda^{2}}{\tau }=\frac{1}{3}\lambda v,$$

where τ is the local mean free time, and we have used that the local swimmer velocity is v=λ/τ. The factor of 1/3 comes from the identity $r^{2}=3r_{i}^{2}$. Using $v=v_{0}{\left(C/C_{0}\right)}^{-1/2}$, we obtain

(9) $$\frac{\partial C}{\partial t}=\frac{v_{0}\lambda C_{0}^{1/2}}{2}\nabla^{2}C^{1/2}\;,$$

or

(10) $$\frac{\partial C}{\partial t}=D_{0}\nabla \cdot \left({\left(\frac{C}{C_{0}}\right)}^{-1/2}\nabla C\right)\;,$$

where $D_{0}=v_{0}\lambda /4$. It may be shown [56] that this equation has the normalizable solution

(11) $$C\left(r,t\right)\propto {\left[y{\left(r,t\right)}^{2}+\frac{8\pi^{4}}{N^{2}}\right]}^{-2}t^{-2}$$

where $y\left(r,t\right)=r/(C_{0}{\left(D_{0}t\right)}^{2})$. At large values of *r*, the solution has a tail $C\left(r,t\right)∼r^{-4}$. For other properties of the solution to concentration dependent diffusivity, see Ref. [56].

### 3.2. Mean Square Displacement and Simulation Results

From the above solution, we can calculate the mean squared displacement $\langle r^{2}\left(t\right)\rangle$ or, equivalently, the root-mean-squared displacement (rms) $r_{rms}\equiv \sqrt{\langle r^{2}\left(t\right)\rangle }$, by the integrals

(12) $$r_{rms}^{2}=\langle r^{2}\left(t\right)\rangle =\frac{\int dVr^{2}C\left(r,t\right)}{\int dVC(r,t)}=\frac{\int_{0}^{\infty }drr^{4}C\left(r,t\right)}{\int_{0}^{\infty }drr^{2}C\left(r,t\right)},$$

where dV=dxdydz is the volume element in three dimensions, and where we changes to radial coordinates in the second equality, with *r* the radius from where the swimmers are released. To extract the leading temporal scaling at late times, we observe that from Equation (11) we have for any exponent α the scaling

(13) $$\int_{0}^{\infty }drr^{\alpha }C\left(r,t\right)∼t^{2+2\alpha }\int_{0}^{\infty }dy{\left[y^{2}+\frac{8\pi^{4}}{N^{2}}\right]}^{-2},$$

where we changed integration variable from *r* to y(r,t), as given above. With this change in the variable, the time-dependence has been made explicit. Using this result together with Equation (12) immediately gives

(14) $$r_{rms}^{2}∼t^{4}.$$

This is a remarkable result, as it predicts a spreading rate which is beyond that of ballistic growth. Ballistic growth, which would result if the swimmers would move at constant speed and never change direction of motion, would give $\langle r^{2}\left(t\right)\rangle ∼t^{2}$.

**Figure 3:**
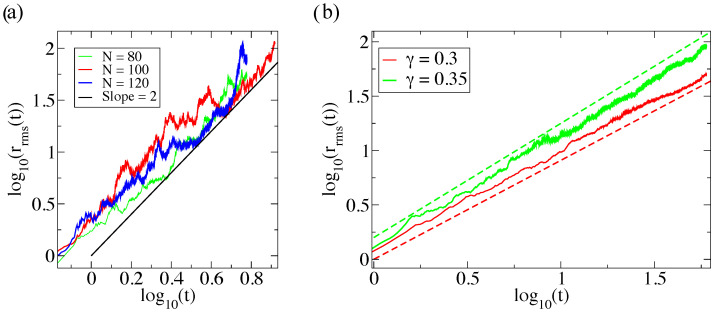
(**a**) The mean square displacement compared to the theoretical values of Equation (14) for different swimmer numbers *N*. The solid line shows the predicted slope, but there is no prediction for the pre-factor in this case. Here, $N_{r}=$ 4. (**b**) The mean square displacement compared to the theoretical values of Equation (15) τ= 0.91 and 1.05 (stapled lines) when γ= 0.3 and 0.35, respectively. Here, N= 100 and $N_{r}=$ 4.

Note that, since $r^{4}C\left(r,t\right)∼r^{0}$ for large *r*, the integral in the numerator diverges. However, as was pointed out in Ref. [40], there will always be a maximal swimmer position $r_{max}$ in any numerical realization, which effectively acts as a cut-off. This means that the proportionality with $t^{4}$ survives and that the prefactor varies linearly with $r_{max}$. Since $r_{max}$ fluctuates in each simulation, the prefactor in Equation (14) is noisy, even with large ensemble numbers. Figure 3a. shows results from numerical simulations where particle concentration was initialized as a delta-peak. The results show that the swimmers indeed conform to the prediction of Equation (14). They are indeed noisy, as expected, since the largest swimmer positions contribute significantly to the $r_{rms}\left(t\right)$ evaluation.

Generalizing the model to $v∼\lambda C^{-\gamma }$ where γ≠1/2, the solution becomes $C\left(r,t\right)∼{\left(y^{2}+k\right)}^{-1/\gamma }$. Now, the integrals in Equation (12) converges when γ<0.4 and, in this case, the $\langle r^{2}\rangle \left(t\right)$ becomes significantly less noisy. The generalized solution leads to $r_{rms}\left(t\right)∼t^{\tau }$, where [40]

(15) $$\tau =\frac{1}{2-3\gamma }.$$

In order to validate both the analytical and computational model, we have carried out simulations using this generalized concentration dependence in the velocity. The results, which are shown in Figure 3b, demonstrate close agreement between simulations and theory.

## 4. Conclusions

We have studied the diffusive behavior of microswimmers competing for resources. Through simple arguments, we have derived a density-dependent Fokker–Planck equation that effectively models this scenario, and have proposed a microscopic numerical model that agrees with the theory. We find that the swimmers exhibit a hyper-ballistic superdiffusive behavior, where the growth of the mean squared displacement in time is quartic. 

## Data Availability

The data presented in this study are available upon reasonable request from the authors.
